# Cloud Digital Forensics: Beyond Tools, Techniques, and Challenges

**DOI:** 10.3390/s24020433

**Published:** 2024-01-10

**Authors:** Annas Wasim Malik, David Samuel Bhatti, Tae-Jin Park, Hafiz Usama Ishtiaq, Jae-Cheol Ryou, Ki-Il Kim

**Affiliations:** 1 Faculty of Information Technology, University of Central Punjab, Lahore 54590, Pakistan; annas.waseem@ucp.edu.pk (A.W.M.); usama.ishtiaq@ucp.edu.pk (H.U.I.); 2 Nuclear System Integrity Sensing & Diagnosis Division, Korea Atomic Energy Research Institute (KAERI), Daejeon 34057, Republic of Korea; 3 Department of Computer Science and Engineering, Chungnam National University, Daejeon 34134, Republic of Korea

**Keywords:** cloud computing, data loss, cloud digital forensic, security breaches, forensic tools, secure data management, compound annual growth rate

## Abstract

Cloud computing technology is rapidly becoming ubiquitous and indispensable. However, its widespread adoption also exposes organizations and individuals to a broad spectrum of potential threats. Despite the multiple advantages the cloud offers, organizations remain cautious about migrating their data and applications to the cloud due to fears of data breaches and security compromises. In light of these concerns, this study has conducted an in-depth examination of a variety of articles to enhance the comprehension of the challenges related to safeguarding and fortifying data within the cloud environment. Furthermore, the research has scrutinized several well-documented data breaches, analyzing the financial consequences they inflicted. Additionally, it scrutinizes the distinctions between conventional digital forensics and the forensic procedures specific to cloud computing. As a result of this investigation, the study has concluded by proposing potential opportunities for further research in this critical domain. By doing so, it contributes to our collective understanding of the complex panorama of cloud data protection and security, while acknowledging the evolving nature of technology and the need for ongoing exploration and innovation in this field. This study also helps in understanding the compound annual growth rate (CAGR) of cloud digital forensics, which is found to be quite high at ≈16.53% from 2023 to 2031. Moreover, its market is expected to reach ≈USD 36.9 billion by the year 2031; presently, it is ≈USD 11.21 billion, which shows that there are great opportunities for investment in this area. This study also strategically addresses emerging challenges in cloud digital forensics, providing a comprehensive approach to navigating and overcoming the complexities associated with the evolving landscape of cloud computing.

## 1. Introduction

**Figure 1:**
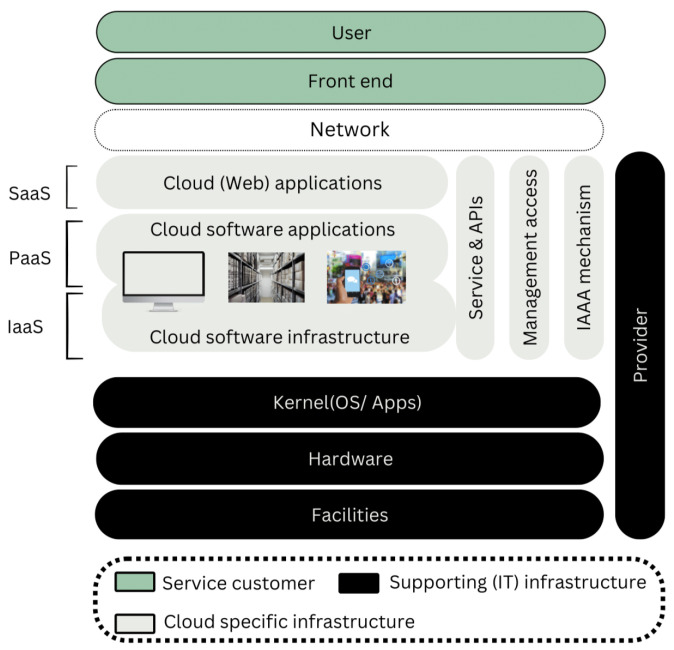
Models of cloud services.

Cloud computing is a framework that permits pervasive, user-oriented, and on-demand admittance to a shared pool of configurable computing assets over the cloud (internet) without direct active management by the user [1]. The primary benefits of cloud computing are not only limited to reduction in time and costs but also agility and scalability. The idea of cloud computing was originally linked to the concepts of distributed parallel computing, utility computing, and autonomic computing. Cloud computing has different models based on deployment and service delivery. Based on cloud deployment, there are four models: public cloud, private cloud, hybrid cloud, and community cloud while based on service delivery; models could be categorized as SaaS (Software as a service), PaaS (Platform as a Service), and IaaS (Infrastructure as a Service), as shown in Figure 1 [2]. Some leading corporations, including Amazon, Google, IBM, Microsoft, Dell Technologies, Hewlett Packard Enterprise, Cisco Systems, and Oracle, have invested in cloud computing and are offering individuals and businesses a range of cloud-based solutions. In the past few years, interest in adopting the cloud computing paradigm has increased not only in the IT industry but also in other sectors like banking, finance, education, health, utility, telecom, etc. According to a study in 2020, the presence of cloud-based applications or computing infrastructure in organizations had increased to 81% from 73% in 2018 [3]. It was forecasted that global end-user investments in public cloud services would grow in 2021 to USD 304.9 billion, up from USD 257.5 billion in 2020 [4]. The ability to use on-demand, adaptable cloud models for achieving cost-effectiveness and business continuity is motivating organizations to rapidly accelerate their digital business transformation plans. Cloud computing is envisioned as a potential future of computing, and there is no doubt that cloud tools and solutions are here to stay. Cloud computing is arguably the most significant technological advancement of the 21st century. However, as cloud computing gains more recognition worldwide, concerns are also being raised about the data security and privacy issues introduced through the adoption of this modern computing paradigm. Data security and privacy have consistently been primary issues in Information Technology. The concerns regarding data security and privacy become particularly serious in the cloud computing environment because data are scattered across various locations on different machines and storage devices, including personal computers, servers, and various mobile devices. Handling data security and privacy in cloud computing is more complex than in conventional information systems. While cloud services are helping remote workers effectively collaborate as part of a team, they are also opening new opportunities for cyber-criminals to conduct cyber frauds. According to a recent study, 92% of the participating organizations still report a cloud security readiness gap, and they are not comfortable with the security consequences of moving their workloads to the cloud environment [5]. According to IBM’s data breach report, the global average total cost of a data breach in the year 2020 was USD 3.86 million with the healthcare sector alone incurring the highest industry cost of USD 7.13 million [6].

In the rapidly evolving digital landscape, data breaches have become a significant concern for organizations across various industries. When a data breach occurs, highly sensitive and confidential information can be compromised, leading to severe repercussions for the affected organization [7]. The aftermath of such incidents can include financial losses, damage to the organization’s reputation, erosion of customer trust, and potential legal consequences. The increasing frequency of data breaches has raised pertinent questions about the security of data stored in cloud computing environments. While cloud computing offers numerous advantages, including flexibility, scalability, and cost-effectiveness, it also introduces inherent security risks [8]. The shared nature of cloud infrastructure and the remote storage of data necessitate a meticulous examination of cloud security practices. Addressing intricate challenges, cloud forensics emerges as a specialized subset of digital forensics, focusing on investigating and mitigating security incidents intrinsic to cloud environments [9,10]. This involves identifying vulnerabilities and attack vectors to facilitate proactive security measures, while also contributing to evidence preservation, incident response planning, regulatory compliance, and the refinement of security strategies. The iterative process sharpens security measures, reinforces employee training, and offers insights for legal remedies and third-party risk management, thus nurturing a resilient and secure digital landscape. Expertise in both digital forensics and cloud technologies is pivotal for this distinctive approach [11]. Proficient practitioners in cloud forensics meticulously gather and maintain evidence in accordance with forensic norms, preserving its integrity and authenticity for potential legal proceedings. The five key phases of digital forensics, which include identification, preservation, collection, analysis, and reporting [12], will be discussed in Section 5.1.

The prevalence and impact of data breaches underscore the criticality of cloud security. While cloud security encompasses measures to protect data and systems from unauthorized access and breaches, it is essential to differentiate cloud forensics within the broader scope of digital forensics. Carrier’s work [13] on file system forensic analysis highlights the distinction between general data security practices and forensic investigations tailored for legal evidentiary standards. Cloud forensics, as a specialized domain within digital forensics, plays a pivotal role beyond data security. It involves investigating incidents, preserving evidence in a manner suitable for court admissibility, identifying vulnerabilities, and facilitating data recovery. Understanding this distinction is crucial, as expert cloud forensics practices are not solely focused on data protection but also on collecting evidence that meets legal criteria. These practices are vital for safeguarding sensitive data, upholding trust in the digital ecosystem, and mitigating the potential fallout of data breaches in cloud computing environments. Cloud forensics analyzes logs, access controls, and user activities to identify vulnerabilities in cloud infrastructure that lead to data breaches [14]. It helps organizations enhance security and recover compromised or deleted data in complex environments [15]. However, experts face technological and legal challenges in cross-border data governance, necessitating collaboration with cloud service providers. Cloud forensics is crucial in investigating incidents, preserving evidence, mitigating fallout, safeguarding sensitive data, and upholding trust in the digital ecosystem [14,15].

### Contributions

The contributions of this paper lie in its comprehensive exploration and analysis of the intricate realm of cloud digital forensics. The article presents an organized framework that delves into not only the fundamental concepts of cloud computing but also the crucial aspects of cloud security and its distinctive relationship with cloud forensics. By thoroughly examining the cloud digital forensic process model, the article highlights the essential stages of identification, preservation, collection, examination, analysis, and presentation, thereby providing a holistic understanding of the complexities involved in this domain. Furthermore, this paper meticulously investigates the challenges associated with cloud forensics, ranging from the identification phase to the presentation phase, shedding light on the intricacies and potential bottlenecks that forensic investigators might encounter. Additionally, the exploration of cloud legal and privacy concerns, along with the projection of the cloud digital forensics compound annual growth rate, further contributes to the comprehensive understanding of the dynamic landscape and its evolving trends. Finally, by identifying open problems and presenting future trends, this paper offers valuable insights into the potential advancements and emerging research directions in the field of cloud digital forensics.

This article is organized as follows: Section 1 presents the introduction; Section 2 focuses on related work; Section 3 explores cloud computing 201; Section 4 discusses cloud services and regulations; Section 5 explores cloud digital forensics; Section 6 explores cloud forensic challenges; Section 7 examines cloud legal and privacy concerns; Section 8 focuses on compound annual growth; Section 9 discusses open research problems; Section 10 focuses on handling emerging cloud digital forensic challenges; and Section 11 presents our conclusions and future work.

## 2. Related Surveys

Cloud computing has notably transformed every segment of our lives and the way of doing business. However, several data protection and security concerns are associated with cloud computing. Many studies have been conducted on data protection and security issues in cloud computing. These research works have emphasized the risks and vulnerabilities in cloud computing and also proposed some solutions, with cloud forensics being one of them. Cloud forensics not only helps in identifying vulnerabilities but also assists in recovering lost data. Ramachandra [16] discussed security implications based on deployment and delivery models in cloud computing. Moreover, he highlighted general vulnerabilities, attacks, and threats, and also proposed some countermeasures like end-to-end encryption and scanning for malicious activities. Mozumder [17] investigated and analyzed real-world cloud attacks and proposed prevention techniques against such malicious activities. M. Ahmed [18] presented a taxonomy of cloud threats. He also described six detailed case studies of cloud data breaches, which demonstrated some of the threats identified in the taxonomy. Furthermore, he applied recent cases of cloud data breaches to the taxonomy to establish whether the taxonomy holds true or not. Srijita Basu [19] covered essential cloud security loopholes in their study and emphasized the importance of understanding these security flaws to devise better countermeasures. The author also conducted a comparative analysis of various cloud security models. One of the many threats to data in the cloud environment is a data breach, which is either an intentional or unintentional disclosure of confidential information to a suspicious environment. Monjur et al. [20] presented a study on cloud data breaches in which they discussed that root factors for a data breach could be both technological and human factors, where most of the time technical factors could be predicted and not human factors as they are dynamic. Since cloud technology delivers on-demand services pertinent to software, platforms, or infrastructure, it is susceptible to numerous types of data breaches. Depending on the kind of data involved, a data breach can result in the destruction or corruption of databases, leakage of classified information, and theft of patents. To track down the potential origin of the data spill, determine what data were compromised, and estimate the total damage or loss caused by the data spill, cloud forensics are needed. Manral et al. [21] presented an extensive survey on cloud forensics classified based on a five-step forensic investigation procedure, discussing in-depth both challenges faced by investigators during cloud forensic investigation and existing cloud forensic solutions. Lei Chen et al. [22] examined novel cloud forensic approaches and tools with the intent to assist cloud forensic experts in forensic investigation procedures in the cloud environment as new threats arise. Khanafseh et al. [10] presented a detailed study on various architectures and solutions in all classes of digital forensics, with a particular focus on cloud forensics. Moreover, they discussed the limitations and drawbacks of existing forensic solutions, providing future research directions. Khan and Varma [23] focused their research on evidence collection and cloud forensic architecture, also implementing a machine learning-based forensic method for the SaaS and PaaS delivery architecture. A fundamental issue often faced by forensic investigators in an investigation is how to carefully and efficaciously collect, preserve, and analyze digital evidence. Fei Ye et al. [24] identified an important challenge that had not been adequately addressed so far in the published literature, that is, the credibility of cloud evidence in a multi-tenant cloud environment. Hence, they proposed a forensics tamper-proof framework (TamForen) for cloud forensics, which could be used in an unreliable cloud environment. The framework depends entirely on the cloud forensics system, independent of the daily cloud activities, implemented on a multi-layer compressed counting bloom filter (MCCBF). Intrusion detection is one of the major concerns in cloud forensics. Sebastian et al. [25] studied the challenges of cybercrimes in rapidly growing cloud computing. Traditional digital forensic methods were insufficient for investigating evidence in cloud platforms. They defined evaluation criteria for digital forensic techniques in IaaS, PaaS, and SaaS models, identifying gaps that require further research. Tummalapalli and Chakravarthy [26] proposed an intrusion detection framework for cloud forensics based on a two-level gravitational group search-based support vector neural network classifier with clustering and a low false-positive rate. Purnaye and Kulkarni [27] proposed a more generic level taxonomy of cloud forensics solutions and strategies that would help researchers gain more knowledge in this field of study. A comprehensive examination was conducted by Alenezi et al. [28] to identify and analyze the prominent challenges encountered in the domains of digital and cloud forensics. The review encompasses a wide spectrum of issues, including data acquisition, analysis, preservation, privacy concerns, and legal complexities. Emphasizing the critical nature of these challenges, this study underscores the imperative to address them effectively, thus ensuring the optimal utilization of digital and cloud forensics in investigative processes.

Table 1 underscores the significant impact of cloud computing on various aspects of life and business while acknowledging the emergence of numerous data protection and security concerns. The studies discussed in this review shed light on the vulnerabilities and risks in cloud computing, prompting the need for specialized cloud forensics and data provenance solutions to address these challenges. Through comprehensive analyses, these research works focused on security issues, cyber-attacks, and countermeasures, particularly within distinct cloud service models. Furthermore, the review highlighted high-profile data breach cases, revealing the urgency to strengthen cloud forensics practices and security measures to combat financial losses and compromised data. It emphasizes the continuous requirement for further research and innovative advancements in the field of cloud forensics to ensure the secure and efficient utilization of cloud computing while mitigating inherent security risks.

## 3. Cloud Computing

**Figure 2:**
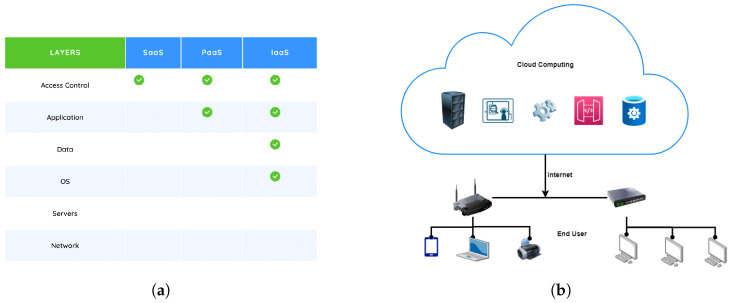
Architecture of cloud computing. (**a**) The variability of customer control levels across various cloud service models. (**b**) Cloud Computing Architecture.

Cloud computing is a revolutionary approach in information technology that leverages the internet to provide on-demand computing resources, transforming how data is stored, accessed, and processed [29]. This paradigm shift eliminates the reliance on local servers, allowing seamless access to applications, storage, and computing power from remote data centers. The three main service models within cloud computing are infrastructure as a service (IaaS), offering high control over infrastructure; platform as a service (PaaS), abstracting control for application development; and software as a service (SaaS), providing minimal control as users access hosted software applications [30]. The control levels of customers vary across different cloud service models, as shown in Figure 2a. Cloud computing, depicted in Figure 2b, empowers organizations and individuals by offering unparalleled scalability, flexibility, and cost-effectiveness. It continues to drive innovation, collaboration, and success in today’s fast-paced, data-driven world while opening up new possibilities for digital transformation, artificial intelligence, and advanced data analytics.

### 3.1. Various Aspects of Data Security and Protection in the Cloud

Cloud security is crucial for businesses relying on cloud computing for essential services like data storage and processing. A robust approach includes strong access controls, encryption techniques, and continuous network traffic monitoring. Proactive patch management, security audits, and vulnerability assessments are essential for maintaining system integrity [31]. As cyber threats evolve, proactive countermeasures like intrusion detection systems and SIEM tools become essential [32]. A well-established cloud security strategy fosters user trust and ensures data protection [33]. Adherence to legal requirements and sector-specific standards, such as HIPAA in healthcare or GDPR in Europe, is also essential for maintaining client confidence in cloud systems [34]. These security aspects are discussed and summarized in Table 2 for quick reference.

**Figure 3:**
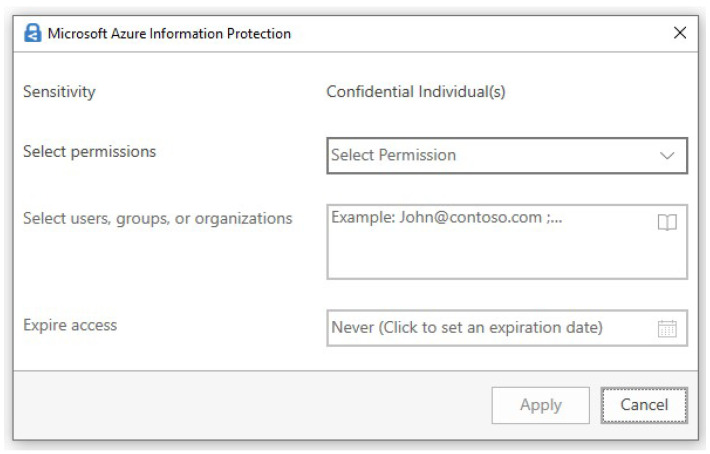
Microsoft Azure information protection.

**Figure 4:**
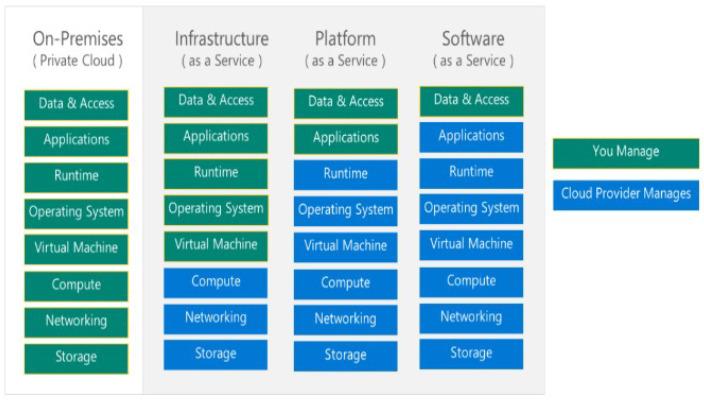
Cloud-shared responsibility model.

1. Security objectives: In cloud computing, data are stored in remote locations, the physical locations of which are unknown and managed by the service provider. The risk factor here is that data may become compromised. Confidentiality is one of the hottest topics these days. Confidentiality means data can only be accessed by authorized users. Preservation of confidentiality increases the trust level of customers in the cloud service providers (CSPs) [35]. Integrity states that there should be no corruption or modification to the data placed in a remote location. Only authorized users and the data owner can recognize that data are in their original form and, after authorized modification, the latest version should be available. This ensures that the data are trustworthy and consistent [36]. Availability ensures that at the time of access, reliable access to the entire data is available for authorized users [37]. Data privacy refers to the extent of information a user wants to share publicly, and private data should remain inaccessible to anyone on the internet [38].
2. Methods to achieve security objectives: Data confidentiality is safeguarded through encryption, where a private key transforms the data into an incomprehensible format during transmission. The security of this process hinges on the complexity of the key, affecting decryption time [39]. In cloud computing, identity-based encryption (IBE) verifies the identities of receivers during decryption for varied data access [40]. Alternatively, attribute-based encryption (ABE) links decryption to specific user attributes, allowing access only if attributes match, thereby enhancing data security [40].
3. Identity and access management (IAM): Identity and access management (IAM) is a security feature in cloud computing that ensures secure access to cloud resources while maintaining the CIA (confidentiality, integrity, and availability) triad. It verifies user identity through federated directory services or directory as a service (DaaS) using SSO (single sign-on), authenticates login using modern authentication features, and provides access based on access rights defined through CSP (cloud service provider) management console [41]. IAM also includes role-based access management (RBAC) and privilege access management (PAM), allowing users to access resources based on their roles and administrative control [42].
4. Information protection: Data are classified based on information sensitivity. For example, if the word salary is detected in any file, then the service provider will automatically mark this file as confidential and process it according to predefined rules. Microsoft offers “Azure Information Protection”, which allows the creation of two types of sensitivity labels: one with predefined rules, so that once a label is selected, the rule is deployed on the file. Another is post-defined, in which the author of the file sets the information protection rule, as shown in Figure 3. The author will enter the email address of the designated recipient, select the permission level (owner, co-owner, read-only, view-only, etc.), and set the expiry date. Figure 3 represents a security label that protects the file, regardless of whether the data server is breached or if the file is moved to unsafe hands. This protection label will allow the file to be opened only by the designated recipients [43].
5. Shared responsibility model: In a local environment, the organization is solely responsible for all types of environmental and data security. However, when infrastructure moves toward a private or hybrid cloud environment, the responsibility is shared between the CSP and the organization’s IT team. Now, both parties work hand in hand to ensure the security of data and infrastructure. Roles are well-defined for the organization’s representatives by the CSP, and data owner rights are duly assigned [44]. Figure 4 represents the cloud-shared responsibility model.
6. Malicious insiders: Insider risk is one of the major data risks nowadays. Competitors may hire such employees or some employees might, for their personal benefits, provide data or their passwords to outside users to access data on their behalf. To mitigate this, security policies like Azure information protection, multi-factor authentication, data classification, etc., are deployed to secure data within organizational boundaries [45].
7. Intentional data remanence: This occurs when data removed from the data servers or cloud data repository reside somewhere in the internal memory or cache, which can be recovered by competitors. CSPs provide this feature to automatically run a removal cycle after a specific period to clear such data from memory [46].
8. Recovery plan objective (RPO): A policy is defined to store a copy of the critical data in a remote location with minimum RTO (recovery time objective). In cases of ransomware or cyber-attacks, when data services go down and data becomes unavailable, CSPs provide some disaster recovery plans, and customization options are also available. Data recovery is dependent on cost, RPO, latency, and geographic separation. Organizational IT representatives, along with other stakeholders, work to reduce these dependencies to achieve maximum RPO with minimum RTO. In case of any incident, a proper incident plan should be followed, and a report must be generated [47].
9. Data segregation/multi-tenant services: CSP service provides a multi-tenancy feature in which multiple copies of data are created and stored at different storage locations. In case of a cyber-attack on one storage location, and it is down, the data will be available to the authorized user from another storage location [48].
10. Data loss prevention: Data loss prevention (DLP) protects sensitive data at rest, in transit, and on endpoints to mitigate the risk of data loss, data theft, and cyber-attacks. The two most significant features are data classification and CASB (cloud access security broker). In data classification, rules are defined based on keywords; when any listed keyword is found in a file, the CSP will process that file according to predefined rules. CASB acts like a proxy server that monitors all activities and implements security policies defined by the CSP. With the emergence of BYOD and the rising aspect of shadow IT, tools like CASB must be implemented to add a security layer for data protection [49,50].

### 3.2. Data Protection Compliance Recommendations

To ensure compliance with data protection authority regulations, organizations should implement the following recommendations or policies [51]: an IAM policy, a disaster recovery plan, a data loss prevention policy, a data encryption policy, an incident response and risk management plan, vulnerability and penetration testing, a data resiliency plan, regular audits, email security, a network defense policy, controlled use of administrative rights, and regular security awareness sessions.

### 3.3. Attacks and Solutions

**Figure 5:**
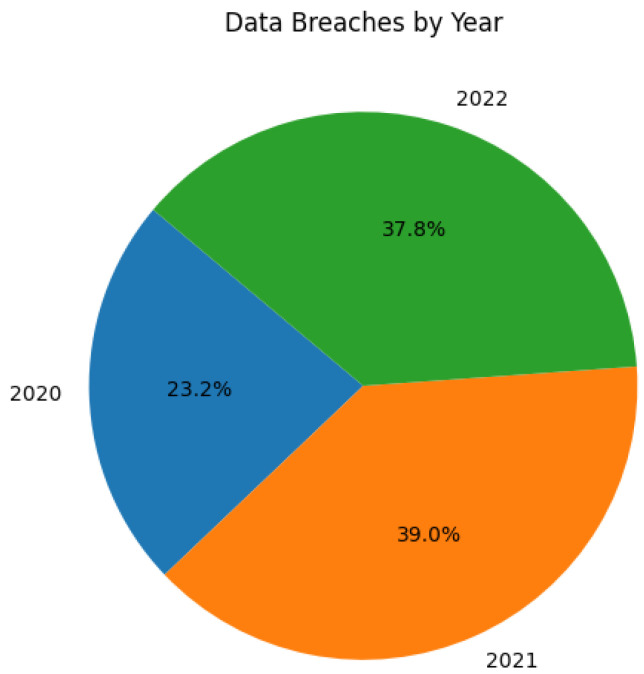
Incidents of data breaches in the cloud environment.

**Figure 6:**
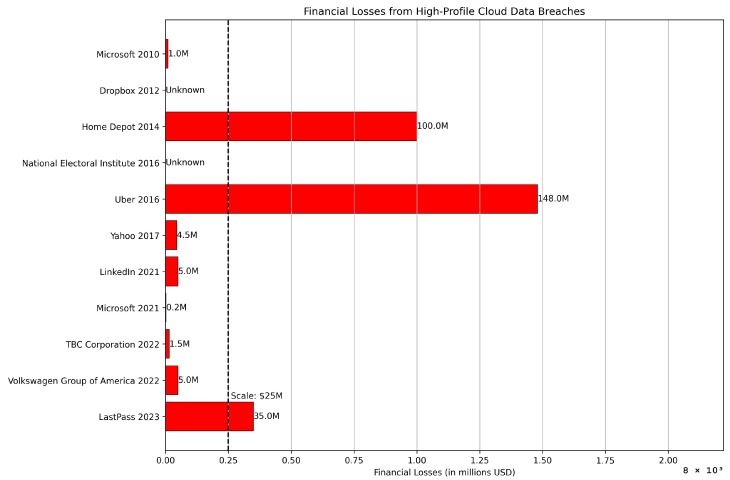
Financial losses from high-profile cloud data breaches.

Data breaches, which can reveal sensitive information to unauthorized parties, have seen a significant increase from 2020 to 2022, with 1108 reported breaches in 2020 and 1862 in 2021. In 2022, there were 1802 breaches, indicating a slight decrease [52], as shown in Figure 5. The 2023 Data Breach Report revealed a significant surge in publicly reported data compromises, with 951 incidents reported in the most recent quarter, a 114% increase from the previous quarter. These statistics highlight the evolving nature of data security challenges in the cloud, requiring increased vigilance and proactive measures to protect sensitive information. Some high-profile data breach cases in the cloud are listed in Table 3 [53,54]:

Financial losses from high-profile cloud data breaches are shown in Figure 6 to understand their global impact on world-class organizations.

To counteract data breaches and security vulnerabilities in a cloud environment, as shown in Table 3, the following solutions are recommended:

1. Data encryption and privacy preservation: Utilize advanced encryption techniques to secure data during transmission and while at rest, rendering sensitive information unreadable and unusable in case of unauthorized access [68]. However, it is vital to acknowledge the limitations of encryption in isolation. The LastPass password manager data breach [67,69] serves as a significant case, demonstrating that encryption, while fundamental, might not guarantee absolute protection. This breach underscores the importance of complementing encryption with robust additional security measures, such as multi-factor authentication, stringent access controls, routine security assessments, and proactive breach response strategies. By integrating encryption within a comprehensive security framework, organizations can enhance their resilience against potential vulnerabilities and address evolving threats more effectively.
2. Access control and identity management: Implement strict access controls based on the principle of least privilege, limiting user access to necessary data and services. Enforce multi-factor authentication (MFA) to add an extra layer of security to user accounts [70,71].
3. Proactive security audits and vulnerability assessment: Conduct regular security audits and vulnerability assessments to identify potential weaknesses promptly. Penetration testing should be employed to simulate real-world attacks and uncover hidden vulnerabilities [72].
4. Timely patch management: Keep software and applications updated with the latest security patches to prevent the exploitation of known vulnerabilities by malicious actors.
5. Real-time security monitoring and incident response: Employ robust monitoring tools and intrusion detection systems to detect abnormal activities early. Establish a comprehensive incident response plan that outlines communication protocols, containment strategies, and recovery techniques.
6. Employee education and training: Continuously educate and train employees in security awareness, familiarizing them with potential threats, phishing attacks, and best practices in data protection.
7. Vendor assessment and compliance: Rigorously assess third-party cloud providers to ensure their security practices, certifications, and compliance align with the framework’s principles [73].

### 3.4. Incident Response in the Cloud

Cloud forensics is crucial in incident response strategies; it involves real-time monitoring and detecting cloud services. It helps organizations identify potential threats, assess the extent of breaches, and gather digital evidence for analysis. Immediate actions are essential to contain the incident, minimize damage, and preserve digital evidence. Key steps to be taken during a cloud security breach include:

1. Isolate affected resources: Swiftly isolate compromised resources within the cloud environment to prevent the breach from spreading further.
2. Alert relevant teams: Notify the incident response team, IT personnel, and pertinent stakeholders to ensure a coordinated response.
3. Collect evidence: Initiate the collection of digital evidence related to the breach, which may involve capturing logs, system snapshots, and network traffic data.
4. Preserve evidence: Maintain the integrity and chain of custody of digital evidence by adhering to best practices in forensic data handling.
5. Forensic analysis: Engage cloud forensic experts to conduct a comprehensive analysis of the collected evidence. This analysis aims to delineate the breach’s scope, pinpoint vulnerabilities, and elucidate the methods and motivations of the attacker.
6. Containment and remediation: Formulate and implement a strategy to contain the breach, remove malicious elements, and remediate vulnerabilities to prevent future incidents.
7. Legal and regulatory compliance: Comply with relevant legal and regulatory obligations, including breach notification requirements that may vary based on jurisdiction and industry.
8. Communication: Maintain open and transparent communication with stakeholders, including customers, partners, and regulatory authorities, providing updates on the incident, its repercussions, and the steps being taken to address it.

### 3.5. Cloud Security vs. Cloud Forensics: Understanding the Distinction

Cloud security and cloud forensics are two distinct domains in the cloud computing world; see [74] and Alenezi, et al. [75]. Cloud security focuses on proactive measures to protect data and resources, including network security, data encryption, and access control. It aims to prevent unauthorized access, data breaches, and potential threats [76]. Incorporating cloud forensics into a comprehensive security strategy is essential to address security threats like data breaches, DDoS attacks, and insider misconduct. Cloud forensics, on the other hand, is a reactive approach that investigates and analyzes incidents, breaches, or unauthorized activities, helping organizations learn from breaches and improve their security posture. Cloud security and digital forensics share similar techniques, but digital forensics strictly adheres to legal guidelines for court admissibility. Privacy laws hold distinct implications, especially when authorized by a judge to scrutinize specific data. In contrast, digital investigation [77] shares methodological similarities with digital forensics but does not necessarily adhere to the same rigorous legal prerequisites for court admissibility. It involves broader inquiries into digital systems, data analysis, and potential security breaches without the stringent legal mandate required for forensic evidence. While digital investigation may not demand identical legal authorization, it remains pivotal to uncovering insights, comprehending incidents, and fortifying organizational security measures. This distinction accentuates the vital role of legal context in digital forensics, ensuring compliance and admissibility within legal frameworks, while digital investigation focuses on thorough exploration and analysis of digital systems without identical legal requisites. Table 4 provides a concise summary, comparing cloud security and cloud forensics.

## 4. Cloud Services and Regulatory Landscape

Organizations from all sectors are increasingly turning to cloud service providers (CSPs) to address their needs for IT infrastructure, data storage, and software, in an era defined by digital transformation. The use of cloud services has reached previously unheard-of levels due to the appeals of cost reductions, scalability, and flexibility. But these changes are also accompanied by a complicated regulatory environment that demands a thorough knowledge of both technology and compliance. In this investigation, we examine how laws and cloud services interact, concentrating on the regulatory bodies in charge of this complex area. Several regulatory bodies around the world play crucial roles in overseeing and shaping the cloud services landscape:

1. European Union Agency for Cybersecurity (ENISA): ENISA is entrusted with enhancing the overall cybersecurity of the European Union. It produces guidelines, recommendations, and best practices to address cybersecurity and regulatory challenges related to cloud services within the EU [78].
2. General Data Protection Regulation (GDPR): While not a regulatory body itself, GDPR is a landmark data protection regulation established by the EU [79]. It has significant implications for cloud services by setting stringent standards for the processing and protection of personal data, even when they are stored or processed in the cloud.
3. National Institute of Standards and Technology (NIST): NIST [80], under the U.S. Department of Commerce, provides a comprehensive framework for cloud computing that covers security, privacy, and interoperability. Their guidelines assist organizations in managing cloud-related risks effectively.
4. International Organization for Standardization (ISO): ISO has developed various standards addressing cloud services, such as ISO/IEC 27017 [81] for security controls and ISO/IEC 27018 [82] for protecting personal data in the cloud. These standards offer a global benchmark for cloud-related best practices.
5. Cloud Security Alliance (CSA): Although not a regulatory body, CSA [83] is an industry association that produces research, tools, and best practices to help organizations address cloud security challenges. Their guidance aids both cloud service providers and users in navigating security concerns.
6. Federal Risk and Authorization Management Program (FedRAMP): Operated by the U.S. government, FedRAMP standardizes the security assessment and authorization process for cloud services used by federal agencies [84]. It ensures that cloud services meet stringent security requirements.
7. Monetary Authority of Singapore (MAS): Notable beyond finance, MAS has issued guidelines on the adoption of cloud services for financial institutions [85]. These guidelines offer insights into managing risks and maintaining regulatory compliance while embracing cloud technology.

A comparison of these regulatory bodies is presented in Table 5.

## 5. Cloud Digital Forensics

Cloud digital forensics is a specialized field that tackles cybercrime investigations in cloud environments, navigating multi-jurisdictional scenarios and evidence preservation protocols [88]. Its complexity is further exacerbated by the concept of multi-tenancy, and the evolving techniques and methodologies employed by cloud forensic experts [89,90].

### 5.1. The Cloud Digital Forensic Process Model

**Figure 7:**
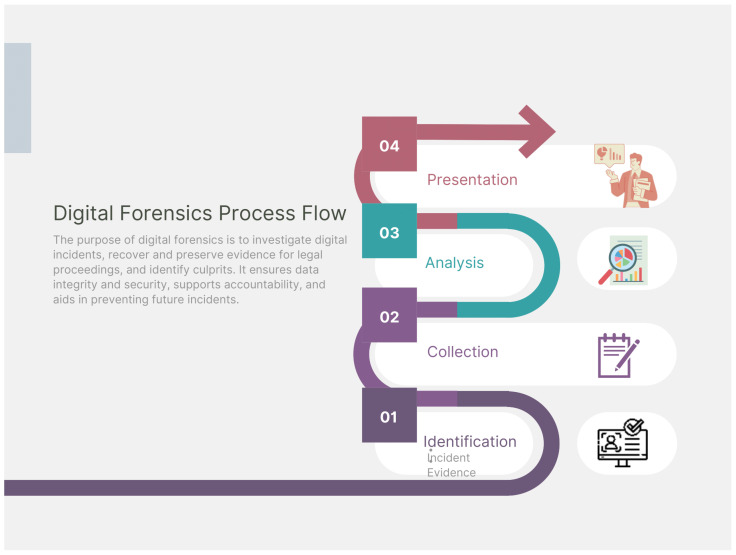
The cloud digital forensics process.

The National Institute of Standards and Technology (NIST) defines digital forensics as a meticulous process that encompasses the recovery, preservation, and analysis of digital data with meaningful applications in criminal investigations and prosecutions [91]. This process is equally applicable to cloud digital forensics, which involves addressing the unique challenges posed by cloud environments. The investigation journey in cloud forensics can be distilled into four pivotal stages [92], each contributing to the comprehensive understanding of a digital incident, as outlined below and depicted in Figure 7. The forensic process consists of the following steps:

1. Identification: Cloud forensics involves identifying and locating relevant cloud-based systems and applications, examining the service provider, services, and data types. Detecting crimes in the cloud is more challenging than traditional forensics, often starting with unauthorized resource usage complaints. New methods are needed to efficiently use existing tools and isolate cloud evidence.
2. Preservation: The preservation stage is crucial for safeguarding digital evidence’s integrity, ensuring its legal use. It involves systematic data capture, secure storage, and documentation, acting as a digital custodian.
3. Examination and analysis: The analysis phase in cloud forensics involves using tools and methodologies to examine digital evidence, uncovering insights through log files, network activity patterns, metadata decoding, and data recovery. This phase requires technical prowess and a discerning eye.
4. Presentation: Cloud forensics aims to present investigative findings in a clear, concise manner, leveraging information as credible evidence in legal proceedings. This involves creating comprehensive reports, using visual aids, and offering expert testimony.

Cloud forensic procedures must adapt to diverse service delivery and deployment models, ensuring the integrity of collected evidence [93]. Rapid evolution of cloud environments necessitates timely capture and retention of evidence to prevent gaps in the evidential trail. Validation of cloud-based evidence in legal proceedings is essential, and techniques like hash codes, digital signatures, and encryption enhance confidence in the veracity of evidence. The robustness of evidence credibility is based on its secure preservation [94].

### 5.2. Cloud Digital Forensics Tools and Technologies

In the realm of cloud digital forensics, the availability of specialized tools plays a pivotal role in facilitating investigations within cloud computing environments. This section offers a comprehensive exploration of prominent cloud digital forensics tools, also listed in Table 6, delineating their key functionalities and significance in uncovering digital evidence.

1. Magnet AXIOM cloud: This tool offers comprehensive cloud data collection and analysis capabilities [95]. It supports various cloud services like AWS, Azure, and Google Cloud, allowing users to recover, examine, and preserve cloud-based evidence.
2. Cellebrite UFED cloud analyzer: The UFED cloud analyzer enables the acquisition and analysis of data from cloud accounts, including social media, email, and storage services [96]. It supports a wide range of cloud providers and helps in uncovering digital evidence.
3. Mandiant CloudLens: This tool by Mandiant, a FireEye company, provides visibility into cloud environments for security purposes [97]. It helps in detecting and investigating threats by monitoring cloud activities and analyzing logs.
4. Volatility framework: Although not exclusively for the cloud, Volatility is a popular open-source memory forensics framework [98]. It is used to analyze memory dumps of virtual machines, including those in cloud environments, to identify signs of compromise.
5. AccessData cloud extractor: This tool facilitates the collection and preservation of digital evidence from cloud storage services, social media platforms, and webmail providers [99]. It assists in building a comprehensive picture of a user’s online activities.
6. AccessData cloud extractor: This tool facilitates the collection and preservation of digital evidence from cloud storage services, social media platforms, and webmail providers [99]. It assists in creating a comprehensive forensic copy of a user’s online activities.
7. Oxygen forensic cloud extractor: Oxygen forensic cloud extractor [100] supports over 20 cloud services, enabling investigators to gather data from cloud storage, social media, and email accounts for digital forensics purposes.
8. Autopsy: While not exclusively designed for cloud forensics [101], Autopsy is an open-source digital forensics platform that allows examiners to analyze evidence from various sources, including cloud storage services.
9. BlackBag BlackLight: BlackLight [102] is a digital forensics solution that supports the analysis of data from both traditional devices and cloud services. It aids in extracting and interpreting data from cloud accounts.
10. X-Ways Forensics: X-Ways Forensics is a versatile digital forensics tool that supports the examination of evidence from cloud storage services, email accounts, and other sources [103].
11. Azure Security Center: Microsoft’s Azure Security Center [104] provides a cloud-native solution for threat protection across Azure and hybrid environments. It helps in detecting and responding to threats in cloud infrastructure.
12. AWS CloudTrail: Amazon Web Services CloudTrail [105] logs all API calls made on an AWS account, allowing for detailed forensic analysis and audit trail creation.

Some other offline digital forensic tools are [106]:

1. EnCase Forensic: EnCase is a widely used forensic software that provides comprehensive capabilities for acquiring, analyzing, and reporting digital evidence from various devices and file systems.
2. AccessData forensic toolkit (FTK): FTK is a powerful forensic tool that allows investigators to collect, analyze, and examine data from computers and mobile devices. It includes advanced searching and analysis features.
3. Forensic Falcon: This hardware-based solution offers both offline and live forensic capabilities, allowing investigators to analyze and image digital media in the field.
4. Paladin Forensic Suite: Paladin is a live forensic system that can be booted from a USB drive. It includes a variety of open-source forensic tools and utilities for evidence collection and analysis.
5. DEFT (Digital Evidence and Forensics Toolkit): DEFT is a Linux distribution specifically designed for digital forensics and incident response. It includes a collection of pre-installed forensic tools and utilities.
6. Bulk Extractor: Bulk Extractor is a command-line tool designed to quickly and efficiently scan disk images for specific types of information, such as email addresses, credit card numbers, and URLs.
7. Digital Forensics Framework (DFF): DFF is an open-source digital forensics platform that provides a modular and extensible framework for conducting forensic investigations.

## 6. Cloud Forensic Challenges

In this section, we provide an overview of the cloud forensics issues observed during the assessment of the relevant domain. Furthermore, we take it a step further and categorize the associated difficulties according to the cloud forensics procedure phases described. It must be noted that the majority of the issues discussed are primarily applicable to public clouds, with only a few exceptions applicable to private cloud designs. These challenges are discussed below, and their summarized view is provided in Table 7 for quick review.

### 6.1. Identification Phase

1. Retrieval of information from log files: Log files are crucial for investigations, but gathering them from cloud computing environments is complex due to cloud haziness and multi-tenant simulations, as clients have access to the application programming interface (API) only, making monitoring impossible [107]. In the IaaS cloud model, logs are essential for understanding virtual machine (VM) behavior, but their effectiveness may be limited due to restrictions imposed by cloud providers on storage, access, or sharing among multiple users [108,109]. Cloud service providers often neglect or conceal log collection services, posing challenges such as decentralization, fluctuation, preservation, accessibility, non-existence, lack of important data, and non-compatible log forms [110].
2. Transient data: Cloud forensic challenges involve navigating the diverse behaviors of virtual machines (VMs) in IaaS service structures, such as Azure, Digital Ocean, and AWS, to preserve data during shutdown or restart phases. Understanding these nuances is crucial for forensic professionals to identify and preserve volatile data instances [111,112,113,114].
3. Lack of physical accessibility: Data localization in the cloud is complex due to the global deployment of hardware equipment. Digital forensics assume direct access to hardware, but cloud forensics struggle due to the storage of information on physical devices and the fixed settings [112]. Data-containing hardware cannot be seized due to dispersed systems in separate jurisdictions. This issue is not relevant for geographically spread firms, where resources are housed on their premises [115].
4. Identification at the client side: Proof can be found on both the supplier and client sides of the interface, particularly in SaaS and PaaS contexts. Investigators must quickly capture sterile data for forensic analysis, as the criminal may destroy it. Client-side data identification is crucial in investigations, but often difficult due to multiple jurisdictions [111,116].
5. Vendor dependency-trust: The research emphasizes the importance of cloud service providers (CSPs) in the forensic process, but challenges arise when they hesitate to release information, especially in multi-tenant systems [117]. Dependence on CSPs in SaaS and PaaS models for evidence discovery raises authenticity concerns and reliance on non-expert personnel, potentially impacting the validity of forensic findings [107,118].
6. SLA (service level agreement: Service level agreements (SLAs) may not include details about forensic investigations, as failure to provide such information can result in a cloud service provider’s lack of contractual obligation [119]. This is often due to a lack of customer understanding, lack of transparency, limits on trust, and foreign legislation. CSPs may not have the necessary knowledge or appropriate procedures to conduct forensic investigations in cloud systems [120].

### 6.2. Preservation and Collection Phase

1. Integrity and stability in multi-tenancy and privacy: The quality and durability of proof are critical in cloud inquiries for IaaS, PaaS, and SaaS. Data retention, essential for evidence in multi-jurisdictional situations, poses challenges in compliance with laws. The reliability of evidence can be compromised, potentially rendering it inadmissible in court [108]. Authenticity issues further complicate cloud forensics, requiring increased trust from investigators in third parties for data authentication [118]. Ensuring data consistency in the dynamic cloud environment is also challenging [121].
2. In-house staffing: This challenge spans all service types and stages, necessitating collaboration among technical researchers, legal consultants, and external experts with expertise in new technologies [120].
3. Crime scene reconstruction in criminal investigations: In cloud forensics, reconstructing the crime scene is challenging, and recreating the entire sequence may be impossible if the responsible virtual machine terminates after malicious activity.
4. Chain of custody: Maintaining the chain of custody is crucial for presenting evidence in court. Challenges arise from multi-jurisdictional legislation and CSP engagement, with the initial potential failure point often identified as the cloud service provider [119].
5. Data imaging: In IaaS, creating a forensic image of a system or instance involves capturing a disk image of the virtual machine (VM) in a defined file format like EWF. Restarting or shutting down the VM does not destroy evidence, but if destroyed, it would be lost. In PaaS environments, relying on the central service provider (CSP) for data collection is crucial, but presents challenges, especially when data are managed by a third-party subcontractor [115].
6. Bandwidth constraints: The amounts of data are rapidly expanding, leading to an increase in evidence. In the preceding paragraph, we discussed VM cloning within the IaaS model. Researchers need to obtain a forensic copy of the VM instances to collect information. While acquiring such extensive data imaging, they have to consider the available bandwidth due to the substantial volume of data involved.

### 6.3. Examination and Analysis Phase

1. Insufficient forensic toolset: In cloud forensic investigations, the use of forensic tools is crucial, with various technologies designed for cloud-based digital forensics actively employed. However, a significant challenge lies in the lack of comprehensive vetting for accuracy and error rates in several commercial tools designed for remote investigations [115]. Initiatives like the computer forensics tool testing (CFTT) program, supported by the Department of Homeland Security (DHS), the National Institute of Justice, and the National Institute of Standards and Technology (NIST), aim to address this gap by providing measurable assurance of the accuracy of computer forensics tools used in cloud investigations [122]. The CFTT program develops specifications and test methods, and evaluates specific tools against these standards to enhance the reliability and credibility of forensic tools. These efforts are crucial for ensuring that forensic tools meet stringent accuracy benchmarks, supporting investigators and the legal community in effectively utilizing these tools within cloud forensic investigations [115].
2. Large data volumes: The data volumes held in CSP storage facilities are enormous and are growing daily. Finding meaningful digital evidence might be complicated by the large amounts of data (petabytes of information) [123]. This has a direct impact on data processing to identify meaningful evidence for the purpose of the inquiry. Quick and Choo [124] further discuss this issue, noting that research gaps in data reduction methods, data mining, intelligence evaluation, and the utilization of open and closed-source information still exists. Appropriate collection and filtering of information must be created and implemented to handle the data quantity that exists in cloud infrastructures [112].
3. Encryption: Cloud clients use encryption to protect against illegal activities. Investigating encrypted material requires expertise in obtaining keys and analyzing content. Accessibility of encryption keys is crucial, and evidence may be undermined if only the data owner can provide the key. Many CSPs also use encryption technologies [125,126].
4. Log format standardization: Analyzing data obtained from service models is a costly operation, particularly when dealing with and identifying a variety of log types. When we are able to access a large number of various resources, combining log forms in the cloud is a complex process [120].

### 6.4. Presentation Phase

1. Password or key retrieval: Cloud forensic investigations encounter distinct challenges, especially in accessing encrypted data without cooperation from involved parties. Advanced tools, such as John the Ripper and Hashcat [127], provide critical support by enabling password retrieval. Additionally, analyzing memory dumps offers avenues for retrieving encryption keys, enhancing investigators’ capabilities to overcome challenges posed by encrypted data in cloud forensic examinations.
2. Testimonial complexity: The complexity of technical details may pose challenges in court comprehension, especially considering that juries typically consist of individuals with minimal understanding of computer systems. Therefore, it becomes crucial for investigators to transparently disclose their methods and procedures [115]. They must be prepared to provide a clear and easily understandable explanation of the cloud, digital forensics, and how they work, as well as clarify how the evidence obtained throughout the inquiry was preserved and recorded. Cloud computing is one of the more complex computer circumstances, and it can stump even the most technically savvy jury. As a result, every piece of evidence must be presented with care, and testimony from experts should be comprehensible to the members of the jury [128].
3. Documentation and record keeping: Another issue is convincing the jury that the proof obtained throughout the investigation has been properly documented and that there had been no modifications to the evidence in prior phases. Researchers must ensure that all parties who participated in the investigation followed methodologies and standards to preserve the chain of custody of the obtained evidence. Electronic documentation encompasses all stages.

## 7. Cloud Legal and Privacy Concerns

Cloud digital forensics is a vital field; it focuses on the investigation and analysis of digital data stored in cloud computing environments, such as those operated by major service providers like Amazon Web Services (AWS), Microsoft Azure, or Google Cloud. This discipline plays a crucial role in uncovering digital evidence, particularly in cases involving cybercrimes, data breaches, or other malicious activities within the cloud [129,130]. One of the primary aspects integral to cloud digital forensics is a clear understanding of the legal and privacy considerations that come into play when dealing with data hosted in cloud infrastructures. When individuals and organizations opt to utilize cloud services for data storage and processing, they effectively entrust their sensitive information to third-party service providers. This scenario prompts important questions concerning data access, its methods, and the specific circumstances under which such access is granted. To delve further into these considerations [28,131]:

1. Data ownership and control: When data are uploaded to the cloud, it is essential to understand that ownership and control can become somewhat blurred. Users technically own their data, but they delegate control over its storage and management to the cloud service provider. This delegation can complicate the process of accessing and analyzing data during a forensic investigation.
2. Access rights: Investigating digital incidents in the cloud requires considering who has access to the data. Cloud service providers typically have physical and administrative access to the servers, and users access their data via web interfaces or APIs. Forensic experts must understand how these access mechanisms work and who has the authority to grant or revoke access.
3. Data encryption and privacy: Many cloud service providers implement robust encryption measures to protect user data. This encryption ensures that even if unauthorized parties gain access to the physical servers, the data remain encrypted and unreadable. While encryption enhances privacy and security, it can pose challenges for forensic investigations, as gaining access to decryption keys may be difficult.
4. Compliance and regulations: Various regions have distinct data protection and privacy regulations. For example, the General Data Protection Regulation (GDPR) [132] in the European Union establishes rigorous requirements for data management and privacy. While conducting investigations in cloud environments, forensic investigators must be mindful of and comply with these regulations. However, it is important to note that when authorized by a court to conduct digital forensics, investigators might operate under legal mandates that supersede certain privacy laws, prioritizing compliance with the court’s directives while maintaining confidentiality and following due legal processes.
5. Cloud service provider policies: Cloud service providers often have their own terms of service and policies regarding data access and disclosure. These policies can impact the process of acquiring data for forensic analysis. Investigators need to be familiar with these policies and work within their constraints.

Incorporating legal considerations into cloud digital forensics involves navigating a wide range of laws and regulations that can vary across different regions. Forensic investigators must prioritize compliance with privacy laws, data protection regulations, and contractual agreements between cloud service providers and users. However, when authorized by a court to conduct digital forensics, practitioners may have different obligations that supersede certain privacy laws, as their actions are mandated by legal authorization and aimed at fulfilling court requirements while ensuring confidentiality and adherence to the legal process.

## 8. Economy Factor: Compound Annual Growth Rate (CAGR)

**Figure 8:**
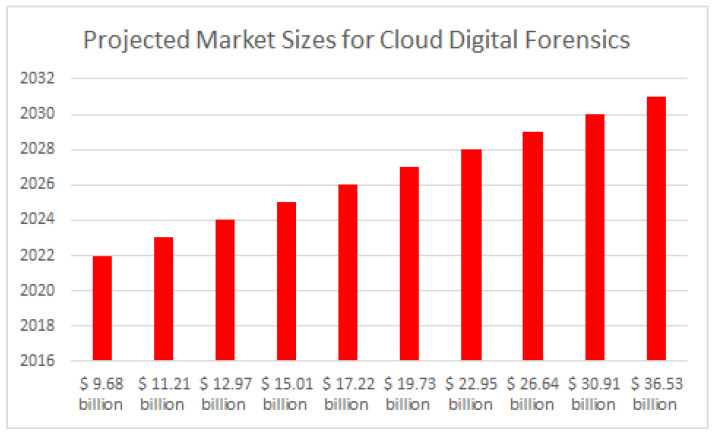
Forecasted growth of cloud digital forensics market (2023–2031).

In the realm of cloud digital forensics, the concept of CAGR plays a pivotal role in understanding and quantifying the sector’s annual expansion. Just as in other industries, CAGR is a vital metric that accurately measures the annual growth of the cloud digital forensics global market. What sets CAGR apart is its ability to account for compounding effects, illustrating how each year’s growth leaves a lasting imprint on the overall trend spanning multiple years. Recent data analysis from market research [133,134] suggests significant growth potential in the global cloud digital forensics market. With a calculated CAGR of 15.9% from 2023 to 2031, the market is expected to witness robust expansion. In 2023, the market size was projected to be around USD 11.21 billion, and is expected to reach USD 36.53 billion by 2031. The data point to a promising upward trend and emphasize the escalating demand for cloud digital forensics solutions over the forecasted period. The graph in Figure 8 visually represents the projected growth trajectory of the cloud digital forensics market from 2023 to 2031, highlighting the anticipated market sizes for each year.

**Figure 9:**
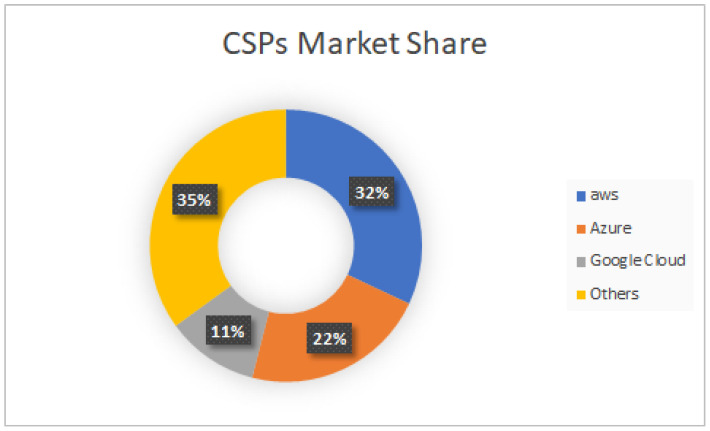
The cloud service provider market share.

This remarkable growth trajectory is, in large part, a response to the escalating incidents of cyber-criminal activities worldwide [135]. These include challenges such as cyber-attacks, industrial espionage, information security breaches, identity fraud, and financial fraud. To address these sophisticated threats, highly skilled digital forensics investigators are at the forefront, working tirelessly to preserve the digital trail of evidence and deliver justice in the digital age. Drawing upon the insights provided by the calculated market values from 2023 to 2031 [133,134,135], industry stakeholders, investors, researchers, and consultants gain a comprehensive understanding of the dynamic growth trajectory within the cloud digital forensics market. Spanning historical data from 2018 to 2022 and extending forecasts up to 2031, these statistics serve as an invaluable reference for current participants and prospective entrants navigating the evolving landscape of cloud digital forensics. Moreover, the current market shares held by prominent cloud service providers have reached unprecedented levels [136]. Projections indicate that major players, including Amazon Web Services (AWS), Microsoft Azure, and Google Cloud Platform, are set to retain their dominance, collectively claiming a significant majority share of the global cloud services market by 2030. The graph in Figure 9 illustrates the market shares of leading cloud infrastructure service providers, providing a visual representation of their current standing in the market. As the demand for scalable and secure cloud solutions continues to surge, the strategic positions of these industry leaders are expected to shape the trajectory of the digital market, driving innovation, and molding the future of cloud computing services.

## 9. Open Problems and Future Trends in Cloud Forensics

Cloud-based digital forensics presents a dynamic landscape with numerous emerging challenges and open issues in the domain of data investigation [137,138]. As businesses progressively embrace cloud services for data storage and processing, safeguarding the security and credibility of digital evidence within intricate cloud infrastructures remains a critical focus. Challenges involve navigating complex multi-tenant environments, tackling concerns about data privacy and sovereignty, and surmounting obstacles stemming from virtualized storage systems and shared resources. The incorporation of sophisticated cryptographic techniques like homomorphic encryption [139] and multiparty computation [140], in conjunction with evolving technologies, such as federated learning [141], introduces fresh hurdles for evidence collection and analysis. Moreover, the assimilation of blockchain-based cloud systems [142] brings forth complexities associated with decentralized data management and the validation of digital transactions. Additionally, ensuring the secure transmission and retention of data across diverse cloud environments while upholding data consistency and integrity persists as significant open challenges [27]. As the cloud landscape continues to evolve, the effective preservation and retrieval of digital evidence, the assurance of a secure chain of custody, and the resolution of intricacies linked with cloud-based data recovery persist as crucial open dilemmas, necessitating continuous research and advancement within the domain of cloud-based digital forensics.

### Future Trends

1. The landscape of cloud digital forensics is continually evolving, and researchers are actively exploring future directions to enhance forensic practices in the cloud. As cloud computing technologies advance, there is a growing need to adapt forensic methodologies to address emerging trends.
2. One key area of exploration is the impact of emerging cloud technologies, such as containerization, microservices, and serverless computing [143], on digital forensics. These technologies introduce new challenges, particularly in the analysis of ephemeral and highly distributed computing environments. Researchers will need to develop techniques to effectively extract and preserve digital evidence in these dynamic settings.
3. Technological advancements, including serverless computing, edge computing, and artificial intelligence (AI), are reshaping forensic practices in the cloud [144]. Serverless computing brings challenges related to event-driven architectures and the reconstruction of execution flows, which researchers will need to address. Edge computing, with its decentralized data processing, requires investigators to adapt to distributed environments. AI, on the other hand, has the potential to automate the detection of security incidents and anomalies, streamlining forensic processes.
4. Advanced cryptographic techniques like federated learning, multi-party computation (MPC), and homomorphic encryption are also influencing cloud and digital forensics [145]. Federated learning enables model training without exposing raw data, posing questions about accessing and analyzing model updates while preserving data privacy. MPC allows secure computations on encrypted data, and homomorphic encryption enables computations on encrypted data without decryption. These techniques introduce both challenges and opportunities for forensic investigators, particularly in scenarios where data privacy is paramount.
5. Blockchain and distributed ledger technologies (DLTs) [146] are gaining prominence in various industries and hold promise for digital forensics. Researchers are exploring how blockchain can be used to create tamper-proof logs and audit trails, enhancing the integrity and traceability of digital evidence. The decentralized nature of DLTs may also influence evidence collection and preservation, ensuring reliability and authenticity.

## 10. Strategizing for Emerging Challenges in Cloud Digital Forensics

The landscape of digital forensics is evolving rapidly with the advent of technologies like the Internet of Things (IoT), cloud-based services (CBSs), cyber-physical systems (CPSs), Blockchain, multiparty computation, federated learning, and the ubiquitous use of mobile devices [147]. Each of these advancements brings its unique set of challenges. IoT solutions introduce a plethora of interconnected devices, amplifying the complexity of data acquisition and analysis. CBSs and CPSs blur traditional boundaries, complicating the identification and preservation of digital evidence spread across diverse platforms. Blockchain technologies pose challenges in tracing and authenticating transactions due to their decentralized and immutable nature. Multiparty computation and federated learning raise concerns regarding data privacy and security, as sensitive information is accessed and utilized across multiple entities. Mobile devices, being an integral part of everyday life, add another layer of complexity due to their mobility, diverse operating systems, and evolving storage methods. Addressing these challenges necessitates proactive strategies that harmonize technological innovation with robust forensic methodologies to ensure effective investigation and resolution in the cloud-based, IoT-driven digital landscape. In this rapidly evolving landscape, navigating the technical challenges of cloud digital forensics requires a versatile toolkit and adaptable strategies. Encountering encrypted files holding crucial evidence often involves a primary but straightforward approach: requesting the password from the suspect. However, in scenarios where collaboration is unattainable, alternative strategies become crucial. Specialized tools like Hashcat and John the Ripper offer avenues for password cracking, presenting intricate solutions to access encrypted data. Integrating these methodologies underscores the importance of leveraging a spectrum of techniques within the evolving cloud-driven digital forensic arena. Moreover, frameworks such as a cloud forensic framework, digital forensic framework, and the application of machine learning principles for forensic methods emerge as essential components. These frameworks focus on data collection, analysis, architecture, and the enhancement of investigation efficiency within cloud environments, addressing challenges specific to different cloud service models. Such a comprehensive approach aligns with the dynamic nature of cloud-based digital forensics, ensuring experts can effectively navigate diverse challenges while upholding ethical and legal standards [23,148].

## 11. Conclusions

Cloud digital forensics is playing an indispensable role in today’s ever-evolving digital landscape. As cloud computing rapidly transforms the information technology (IT) landscape, it is crucial to understand its profound impact on digital forensics, affecting various stakeholders, from forensic investigators and equipment vendors to law enforcement agencies and corporate compliance and audit departments. With the increasing cross-national nature of cloud services, complexities arising from jurisdictional discrepancies and diverse data protection laws demand a refined approach from digital forensic specialists. Successful navigation of this complex regulatory landscape is essential to ensure both legal adherence and the safeguarding of individuals’ privacy in the digital sphere. The integration of artificial intelligence (AI), edge computing, and advanced cryptography into cloud environments presents both opportunities and challenges. AI can aid in automating certain forensic tasks and detecting anomalies, but it also introduces new vulnerabilities that forensic experts must address. Similarly, the use of blockchain and distributed ledger systems can enhance the integrity of digital evidence. Making use of these technologies offers tamper-proof data storage and verifiable chains of custody, providing a robust solution for preserving and presenting digital evidence in court. Collaborative research among stakeholders is needed to develop new techniques, tools, and best practices for cloud forensics, one of the growing fields. The promising investment prospects within the global cloud forensics industry have been clearly evidenced by the CAGR in 2023, which is ≈USD 11 billion, and is expected to reach ≈USD 36.53 billion in 2031.

## Figures and Tables

**Table 1:** Related studies on cloud forensics.

Sr. No	Authors	Breaches	Tools	Challenges	Security Aspects	Legal and Privacy Concerns	CAGR
1	Ramachandra [16]	✓	X	✓	✓	✓	X
2	Mozumder [17]	✓	X	X	✓	X	X
3	M. Ahmed [18]	✓	X	✓	X	X	X
4	Srijita Basu [19]	✓	X	✓	✓	X	X
5	Monjur et al. [20]	✓	X	✓	X	X	X
6	Manral et al. [21]	✓	✓	✓	✓	✓	X
7	Lei Chen et al. [22]	✓	✓	✓	✓	✓	X
8	M Khanafseh et al. [10]	✓	✓	✓	✓	X	X
9	Y Khan and S Varma [23]	✓	✓	✓	✓	X	X
10	Fei Ye et al. [24]	✓	✓	✓	✓	✓	X
11	Sebastian et al. [25]	✓	✓	✓	✓	✓	X
12	Tummalapalli and Chakravarthy [26]	✓	✓	✓	X	X	X
13	Purnaye and Kulkarni [27]	✓	✓	✓	X	X	X
14	Alenezi et al. [28]	✓	✓	✓	✓	✓	X
15	**Proposed**	✓	✓	✓	✓	✓	✓

**Table 2:** Summary of various aspects of data security and protection in the cloud.

Sr. No.	Aspect	Description
1	Confidentiality	Data access restricted to authorized users.
2	Integrity	Data remains uncorrupted and in its original form.
3	Availability	Reliable access to data for authorized users.
4	Privacy	Protection of private data from unauthorized access.
5	Data encryption	Use of encryption for confidentiality and privacy.
6	Identity and access management (IAM)	Secure access to cloud resources, including authentication and access rights management.
7	Information protection	Classification and protection of sensitive data.
8	Shared responsibility model	Distribution of security responsibilities between CSP and organizations.
9	Malicious insiders	Mitigation of insider data risks.
10	Intentional data remanence	Secure removal of data from storage.
11	Business continuity plan	Data backup and recovery strategies.
12	Data segregation/multi-tenant services	Multiple copies of data in different storage locations.
13	Data loss prevention (DLP)	Protection against data loss and theft.
14	Data protection compliance recommendations	Policies for regulatory compliance.

**Table 3:** High-profile data breach cases in the cloud.

Year	Organization	Vulnerability	Data Loss	Financial Loss
2010	Microsoft [55]	A configuration issue within its business productivity online suite (BPOS)	Employee contact data for a small number of users were stolen.	Around USD 1 million
2012	Dropbox [56]	End users and their security settings	A total of 68 million user accounts were hacked	Unknown
2014	Home Depot [57]	An attack exploited the Home Depot’s point-of-sale terminals	Information from 56 million credit cards was stolen	Over USD 100 million
2016	National Electoral Institute of Mexico [58]	Unsecured data were published online	A total of 93 billion voter registration records were compromised	unknown
2016	Uber [59]	Vulnerable Creepy Stalk version	57 million users’ data and 60 million drivers’ license information were exposed	USD 148 million
2017	Yahoo [60]	Session Hijack	3 billion user accounts hacked	USD 4.5 million
2021	LinkedIn [61]	Network Scraping	A total of 700 million user accounts posted for sale on the dark web	USD 5 million
2021	Microsoft [62]	The breach occurred due to a misconfiguration in one of Microsoft’s cloud databases, which left the data exposed without proper access controls	Sensitive data of over 38 million Microsoft users were exposed, including email addresses, account IDs, and support case details	$ unknown
2022	TBC Corporation [63]	Misconfigured AWS S3 Bucket	Approximately 17,000 customer records, including personally identifiable information (PII), such as names, addresses, and phone numbers	Est. USD 1.5 million
2022	Volkswagen Group of America [64]	Exposed Elasticsearch cluster	Over 3.3 million records, including customer information and internal data, were exposed. The exposed data included employee names, email addresses, and some customer data	Est. USD 5 million
2023	Microsoft Cloud [65]	Forged authentication tokens	It primarily targeted government agencies in Western Europe and focused on espionage, data theft, and credential access	unknown
2023	LastPass [66,67]	Targeted attack on a DevOps engineer’s home computer using a vulnerability in the Plex media server package.	Obtained password vaults with encrypted and plaintext data from 25 million users. Exposed seed phrases used for cryptocurrency investments, leading to significant theft	USD 35 million worth of crypto

**Table 4:** Comparison between cloud security and cloud forensics.

Aspect	Cloud Security	Cloud Forensics
Focus	Proactive measures and strategies to safeguard data and resources stored in the cloud	Reactive approach, investigating and analyzing incidents, breaches, or unauthorized activities within the cloud after they have occurred.
Key objective	Prevent unauthorized access, data breaches, and potential threats	Investigate incidents, understand their nature and extent, and enhance overall security readiness.
Key components	Cloud security involves network security measures like firewalls, robust data encryption protocols, and access control mechanisms to protect data at rest and in transit, ensuring a secure cloud environment.	Cloud forensics uses specialized tools for digital evidence collection and analysis, including software, data acquisition, and data interpretation, to reconstruct events in security incidents, enabling investigators to reconstruct the sequence of events.
Role in incident response	Cloud security plays a critical role in establishing a robust defense mechanism to prevent security incidents and breaches. It focuses on proactive measures to minimize the likelihood of incidents occurring in the first place.	Cloud forensics is crucial in incident response, identifying the root causes of security incidents, holding responsible parties accountable, and implementing preventive measures. It collects and analyzes digital evidence post-incident.
Typical activities	Implementing security layers, including network security, data encryption	Collecting and analyzing digital evidence, post-incident analysis.
Expertise required	Security professionals, network administrators	Digital forensic analysts, incident responders
Time frame	Ongoing process to maintain security	Typically initiated after a security incident occurs

**Table 5:** Comparative analysis of cloud regulatory bodies.

Regulatory Body	Geographical Focus	Key Regulations	Compliance Requirements	Certification Programs	Enforcement
GDPR [79]	European Union	Data Protection, Privacy Rights	Consent Management, Data Breach Notification	GDPR Certification	Fines up to 4% of global turnover
HIPAA [86]	United States	Healthcare Data Privacy, Security Standards	Protected Health Information (PHI) Safeguards	HIPAA Compliance Certification	Fines up to USD 1.5 million per violation
ISO/IEC 27001 [87]	International	Information Security Management	Risk Assessment, Security Controls	ISO/IEC 27001 Certification	Audits and Certifications
FedRAMP [84]	United States	Cloud Service Providers (CSPs) for Federal Agencies	Security Controls, Continuous Monitoring	FedRAMP Authorization	Ongoing Assessments, Authorization Reviews
CSA STAR [83]	International	Cloud Security, Risk Management	Security Controls, Transparency	CSA STAR Certification	Self-assessment and Third-party Audit
ENISA [78]	European Union	Cybersecurity Guidelines, Best Practices	Compliance Frameworks, Regulatory Challenges	-	Guideline Adherence
NIST [80]	United States	Cloud Framework (Security, Privacy, Interoperability)	Risk Management, Compliance Measures	-	Guideline Adherence
MAS [85]	Singapore	Cloud Guidelines for Financial Institutions	Risk Management, Regulatory Compliance	-	Financial Compliance

**Table 6:** Summary of digital forensic tools and their features.

Category	Tools	Features
Cloud digital forensic tools	Magnet AXIOM cloud	Comprehensive cloud data collection and analysis
	Cellebrite UFED cloud analyzer	Acquisition and analysis of data from cloud accounts
	Mandiant CloudLens	Visibility into cloud environments for security
	Volatility Framework	Memory forensics framework for virtual machines
	AccessData cloud extractor	Collection and preservation of digital evidence
	Oxygen forensic cloud extractor	Supports over 20 cloud services for forensics
	Autopsy	Open-source digital forensics platform
	BlackBag BlackLight	Analysis of data from devices and cloud services
	X-Ways Forensics	Examination of evidence from cloud storage, email, etc.
	Azure Security Center	Threat protection in Azure and hybrid environments
	AWS CloudTrail	API call logs in AWS accounts for forensic analysis
Offline digital forensic tools	EnCase Forensic	Comprehensive forensic software for evidence
	AccessData Forensic Toolkit (FTK)	Tool for collecting, analyzing, and examining data
	Forensic Falcon	Hardware-based solution for offline and live forensics
	Paladin Forensic Suite	Live forensic system bootable from a USB drive
	Digital Evidence and Forensics Toolkit (DEFT)	Linux distribution for digital forensics
	Bulk Extractor	Command-line tool for scanning disk images
	Digital forensics framework (DFF)	Open-source digital forensics platform that provides a modular and extensible framework for conducting forensic investigations.

**Table 7:** Summary of challenges and recommendations for cloud digital forensics in different phases.

Phases	Challenges	Recommendations
Identification	Retrieval of information from log files Transient data Lack of physical accessibility Identification at the client side Vendor dependency–trust SLA (Service level agreement)	Implement robust logging mechanisms in cloud environments. Develop procedures for handling transient data and capturing it before shutdown or restart. Advocate for standardized access to physical infrastructure in cloud service agreements. Emphasize client-side data identification and preservation. Encourage transparency and cooperation between CSPs and investigators. Ensure SLAs include forensic investigation protocols.
Preservation	Integrity and stability in multi-tenancy and privacy In-house staffing Crime scene reconstruction in criminal investigations Chain of custody Data imaging Bandwidth constraints	Develop encryption and privacy-preserving techniques for multi-tenancy. Build multidisciplinary teams for cloud forensics investigations. Explore innovative methods for reconstructing cloud-based crime scenes. Establish a clear chain of custody protocols in cloud investigations. Create standardized procedures for data imaging in various cloud service models. Consider high-speed data transfer solutions for handling large volumes of data.
Examination and Analysis	Insufficient Forensic Toolset Large volume of data Encryption Log format standardization	Invest in the development and validation of specialized forensic tools for cloud environments. Explore data reduction and analysis techniques for handling vast amounts of cloud data. Develop expertise in encryption key management and legal decryption methods. Promote log format standardization across cloud service providers.
Presentation	Testimonial Complexity Documentation and record keeping	Train forensic experts to simplify technical explanations for non-technical audiences. Maintain meticulous records and documentation throughout the investigation process.

## Data Availability

Not applicable.
